# Associations Between Neurofeedback, Anthropometrics, Technical, Physical, and Tactical Performance in Young Women’s Football Players

**DOI:** 10.3390/jfmk10040423

**Published:** 2025-10-30

**Authors:** Sílvio A. Carvalho, Pedro Bezerra, José E. Teixeira, Pedro Forte, Rui M. Silva, José Mª Cancela-Carral

**Affiliations:** 1 Faculty of Educational Sciences and Sports Sciences, University of Vigo, 36005 Pontevedra, Spain; silvio.carvalho@live.com.pt; 2 SPRINT—Sport Physical Activity and Health Research & Innovation Center, 4900-347 Viana do Castelo, Portugal; pbezerra@esdl.ipvc.pt (P.B.); jose.eduardo@ipg.pt (J.E.T.); ruimiguelfps@hotmail.com (R.M.S.); 3 Department of Sports, Higher Institute of Educational Sciences of the Douro, 4560-708 Penafiel, Portugal; pedromiguel.forte@iscedouro.pt; 4 School of Sports and Leisure, Polytechnic Institute of Viana do Castelo, 4900-347 Viana do Castelo, Portugal; 5 Sports Science Department, Polytechnic of Cávado and Ave., 4750-810 Guimarães, Portugal; 6 Sports Science Department, Polytechnic Institute of Guarda, 6300-559 Guarda, Portugal; 7 CI-ISCE, ISCE Douro, 4560-708 Penafiel, Portugal; 8 Research Center for Active Living and Wellbeing (Livewell), Instituto Politécnico de Bragança, 5300-253 Bragança, Portugal; 9 Sports Science Department, Instituto Politécnico de Bragança, 5300-253 Bragança, Portugal

**Keywords:** neurofeedback, soccer, performance, decision-making, motor skills

## Abstract

**Background**: Neurofeedback training has emerged as a promising tool for enhancing performance by targeting specific brain activity patterns linked to motor skills, decision-making, and concentration. This study aimed to explore the associations between neurofeedback outcomes and football-specific performance metrics, including anthropometric, physical, technical, and tactical dimensions. **Methods**: A quasi-experimental design was used to examine the effects of a six-week neurofeedback training program on motor skills, tactical decision-making, and physical performance in young women’s football players (*n* = 8, aged 14–18). Participants underwent 30-min sessions three times a week targeting sensorimotor rhythms (SMRs) in the 12–15 Hz range within virtual football scenarios. Pre- and post-intervention assessments included anthropometric measures, neurophysiological evaluations, Loughborough Soccer Shooting Test (LSST), and Yo-Yo Intermittent Recovery Test Level 1 (YYIR1). Tactical decision-making was evaluated with a FUT-SAT-based instrument, and biological maturity was estimated using the Mirwald equations. **Results**: Statistical analyses using Pearson’s correlations revealed significant associations between neurofeedback outcomes, motor efficiency indices (MEIs), decision-making (DM), and football performance metrics. Correlation coefficients ranged from 0.504 to 0.998, with *p*-values from 0.010 to <0.001, indicating significant associations across physical, technical, and tactical dimensions. **Conclusions**: This study highlights the beneficial impact of neurofeedback on football performance in young female athletes.

## 1. Introduction

Neurofeedback training has emerged as a promising tool to enhance cognitive and motor performance in sport contexts [1,2,3]. By providing real-time information on brain activity, neurofeedback allows athletes to modulate neural states associated with attention, arousal regulation, and executive control [4]. This modulation is particularly relevant in football, characterized by complex, rapidly changing environments that require efficient decision-making, tactical adaptability, and technical precision [3]. Previous research has demonstrated that neurofeedback can enhance cognitive processes such as sustained attention, working memory, and perceptual sensitivity, which are essential for optimal tactical performance during competition [5]. These skills must be executed under high-pressure conditions, particularly in youth players who are navigating the tumultuous period of adolescence [6,7].

Understanding the mechanisms that contribute to football performance is crucial for optimizing training protocols and fostering player development [8]. However, the application of neurofeedback in young women football players remains an area that warrants further exploration and research [9,10]. Based on this, new contributions are essential study intends to present new contributions for neurofeedback analysis in the performance analysis of youth women football. First, it focuses specifically on youth women football, a population largely underrepresented in the neurofeedback literature, thereby addressing an important gap in current scientific knowledge [11,12]. Second, it integrates neurofeedback outcomes with tactical performance indicators, offering an innovative perspective on the relationship between neurophysiological processes and tactical behavior in football [13,14]. Third, it employs a multidimensional correlational approach, combining neurofeedback measures with multiple football-specific performance dimensions to provide a more comprehensive understanding of the interactions between neural and performance-related variables. Neurofeedback using target sensorimotor rhythms (SMR) develops improvement strategies in different psychophysiological strategies such as focus, motor efficiency, and decision-making [3,15]. It enhances motor imagery, attention, and task execution, vital for physical, technical, and tactical skills [16,17]. For young women players, the neurofeedback could bridge cognitive adaptability with on-field performance, complementing traditional training to optimize skill development [18,19]. Despite its potential, neurofeedback remains underexplored in this demographic, highlighting the need for tailored interventions to address their specific developmental challenges [20,21].

Additionally, physical fitness and technical abilities are foundational to football performance. Aerobic endurance, for instance, is critical for sustaining high-intensity actions throughout a match and is often assessed using the Yo-Yo Intermittent Recovery Test Level 1 (YYIR1) [22]. This test provides valuable insights into a player’s capacity to maintain performance levels during the physically demanding nature of football. Additionally, technical skills such as passing, dribbling, and shooting are essential for both individual and team success. The Loughborough Soccer Shooting Test (LSST) serves as a reliable measure for evaluating these technical abilities, highlighting the importance of integrating physical conditioning with technical proficiency in performance assessments [22,23,24]. The interplay between these aspects enables players to execute precise actions even when fatigued, emphasizing the necessity of a holistic approach in training and evaluation [18]. Also, anthropometric characteristics play a crucial role in football performance. These measures are not only linked to strength, speed, and endurance capacities, but also interact with technical efficiency and tactical execution during play [7,25]. In addition, anthropometric variables are essential to estimate biological maturation, which is a critical determinant in adolescence, affecting players’ capacity to adapt physically and cognitively to training demands. Previous studies have shown that growth- and maturation-related differences influence aerobic performance, neuromuscular development, and decision-making processes in youth football players [26,27]. For this reason, anthropometric evaluation provides a fundamental baseline to contextualize physical, technical, and tactical performance in young women football players. Tactical proficiency in football encompasses decision-making, positioning, and the ability to balance offensive and defensive responsibilities. Metrics such as the Motor Efficiency Index (MEI) and Decision-Making (DM) provide quantitative insights into a player’s capacity to optimize space, disrupt opponents, and maintain tactical equilibrium during gameplay. Tools like the FUT-SAT assess players’ effectiveness in applying tactical principles in match-like scenarios, underscoring the cognitive demands inherent in football [28,29,30]. A comprehensive understanding of how players navigate their tactical roles while integrating physical and technical actions is vital for fostering overall performance development [30].

Integrating neurofeedback with conventional methods could provide holistic training programs, advancing both cognitive and physical performance while fostering a balanced approach to skill acquisition [31]. Young and adolescent players show notable differences in technical, tactical, physical, and cognitive domains due to growth, maturation, and training experience. Physically, aerobic and anaerobic capacities, strength, and speed increase but with large variability [25]. Technically, the refinement of core skills progresses with practice, though still influenced by coordination and motor control linked to maturation [18]. Tactically, the young women football players demonstrate greater ability to adapt positioning, anticipate actions, and balance offensive and defensive roles [28]. Cognitively, advances in executive functions and decision-making support more complex responses in game situations [32,33]. These developmental variations highlight the need to consider age and maturity when analyzing adolescent football performance. Considering maturation allows a more accurate interpretation of technical, physical, and tactical performance, since athletes of the same chronological age may be at different stages of biological development [34]. This variable is therefore essential to contextualize these insights in youth women football.

Indee, the previous studies on neurofeedback in women’s football are practically non-existent, especially when considering the improvement of technical and tactical skills. The application of this methodology can serve as complementary training in the technical and tactical development of skills that can then improve individual and collective performance, but it can also enable eye-foot cognition training during prolonged periods of injury or during congested periods when recovery needs overlap with effective training. Thus, this study aims to investigate the associations between neurofeedback outcomes and football-specific performance metrics, including anthropometric, physical, technical, and tactical dimensions. The central hypothesis is that neurofeedback training targeting SMR will positively correlate with improvements in motor efficiency, decision-making, and overall football performance. By addressing this gap, the research seeks to advance the understanding of how neurofeedback can enhance athletic development in young women’s football players.

## 2. Materials and Methods

### 2.1. Study Design

This observational cross sectional was integrated in a quasi-experimental design with a pre- and post-intervention comparison to investigate the associations with neurofeedback-monitored data from 6 weeks on young female football players’ motor skills, tactical decision-making, and physical performance. Participants underwent neurofeedback sessions targeting SMR in the 12–15 Hz range. The study involved a baseline assessment phase, a six-week monitoring with three sessions per week. Each session lasted approximately 30 min and included visualization of football-specific scenarios and real-time neurofeedback tasks using virtual environments designed to simulate game conditions. Pre- and post-intervention evaluations included anthropometric measures, neurophysiological assessments, and performance tests such as the LSST and YYIR1. Tactical decision-making was evaluated using a FUT-SAT-based instrument, and maturity status was calculated using the Mirwald equations. The assessments were performed twice: at baseline (week 0) and post-intervention (week 6). Neurofeedback sessions occurred three times per week with at least 48–72 h between sessions, always under standardized conditions (same training facility, afternoon hours, controlled temperature, and after 24 h without strenuous exercise). After that, associations of neurofeedback mean scores and physical, technical, and tactical dimensions were possible to output.

The inclusion criteria were as follows: (i) women football players competing in youth federated teams (U15–U19); (ii) a minimum of three years of structured football training and competitive experience; (iii) voluntary participation with signed informed consent (and parental authorization when applicable); (iv) absence of musculoskeletal or neurological injuries within the previous six months that could affect physical, technical, or cognitive performance; and (v) attendance in at least 80% of the experimental sessions, including neurofeedback, anthropometric, technical, physical, and tactical assessments. The exclusion criteria were: (i) any diagnosed neurological, psychiatric, or epileptic disorders incompatible with neurofeedback procedures; (ii) regular use of medications, stimulants, or supplements that could influence cortical activity or performance outcomes; (iii) occurrence of musculoskeletal injury or concussion during the experimental period or within six weeks prior to testing; (iv) failure to complete the neurofeedback protocol or the scheduled physical, technical, or tactical evaluations; and (v) incomplete or inconsistent data, such as EEG signal loss or missing anthropometric or performance records.

### 2.2. Sample

The study included youth female football players aged 14 to 18 years, recruited from competitive teams affiliated with local football academies (Table 1). A total of 8 participants were selected based on the inclusion criteria, which required a minimum of three years of organized football experience, regular participation in competitive matches, and at least three weekly training sessions. Participants were segmented into six groups, with group assignments following the sequential order of assessments. Each group was evaluated only after the completion of the preceding group. Players with any recent injuries, neurological conditions, or contraindications for neurofeedback training were excluded. The sample had an average age of 16.2 years (±1.1), an average height of 162.5 cm (±5.8), and an average body mass of 58.3 kg (±6.9). Participants and their legal guardians provided informed consent, and ethical approval was obtained from the institutional review board prior to the study (CECSVS2023/11/viii). The study followed the recommendations of the Helsinki Declaration regarding human research.

### 2.3. Procedures

#### 2.3.1. Anthropometrics and Physical Fitness

Body measurements, including body mass, standing height, and seated height, were performed by qualified professionals to ensure precision. The procedures and materials outlined below align with contemporary standards and recommendations [35,36]. To measure body mass, a digital scale with an accuracy of ±0.1 kg is preferred (Tanita Corporation, Tokyo, Japan). A stadiometer mounted on a flat wall is recommended for standing height assessments, ensuring measurements are taken on a level, solid surface (Seca, Hamburg, Germany). For seated height and other dimensions, a flexible, non-elastic measuring tape is required. All measurements must adhere to standardized protocols to maintain reliability and validity.

Estimating biological maturity was conducted using validated predictive models tailored to youth populations. The Mirwald equations, commonly applied, estimate the maturity offset, defined as the number of years a child is from reaching their peak height velocity (PHV) [37]. Precise assessments of standing height, seated height, and body mass are critical for accurately applying these equations. Standing height is measured while the individual stands erect without shoes, ensuring the head is aligned in the Frankfort horizontal plane. Seated height is determined by measuring from the seating surface to the top of the head, with the individual sitting upright on a flat surface. Body mass is recorded with participants wearing light clothing and no footwear. Negative values indicate the years before PHV, while positive values reflect the years after PHV. Maturity timing was categorized into groups based on z-scores: values greater than 0.5 indicated early maturity, scores between −0.5 and 0.5 signified average maturity (indicating the individual was within the typical maturity range), and scores below −0.5 represented delayed maturity. These categorizations were based on validated references [25].

#### 2.3.2. Neurofeedback

The six-week neurofeedback protocol for youth female football players focused on enhancing motor skills and tactical decision-making through targeted interventions using electroencephalography (EEG)-based neurofeedback systems [13]. Participants engaged in three sessions per week, each lasting approximately 30 min, where SMR in the 12–15 Hz range were targeted via full-cap EEG-based system with real-time feedback through visual interface. Full-cap EEG was used, but analysis focused on SMR at C3/C4. The EEG neurofeedback was chosen for ecological validity, faster setup in sports settings, and reduced discomfort for adolescents, despite lower signal-to-noise ratio compared to wet systems. Training included visualization of football-specific scenarios to improve motor imagery and decision-making. Sessions began with baseline assessments to establish individual brain activity patterns, followed by real-time neurofeedback tasks using virtual environments that simulated game-specific challenges, such as accurate passing, shooting, and defensive positioning. Feedback was provided visually through a computer screen to guide participants in modulating SMR activity. The protocol incorporated progressive difficulty, starting with static tasks and advancing to dynamic challenges mimicking competitive game scenarios. Researchers ensured individualized adjustments based on performance data to optimize neuroplasticity and motor skill development. This protocol was grounded in evidence from motor imagery and neurofeedback studies, showing improved cortical and corticospinal tract excitability [38]. Ethical considerations were addressed, and informed consent was obtained from all participants and guardians. Data were analyzed to assess changes in neurophysiological activity, skill performance, and decision-making quality over the intervention period.

Regarding the duration of each session, our recommendation is to allocate approximately 30 min in total, including 5 min for preparation, 5 min for calibration, and 20 min for effective training. After completing the first training session, we would like to further clarify how the difficulty levels should be adjusted. For each parameter—power and accuracy—the number of stars can be selected, with each star representing a difficulty level from 1 to 5 (1 being the lowest and 5 the highest). The objective is to ensure that each player trains at the appropriate difficulty level to achieve the greatest impact, similar to selecting the correct weights in a gym setting. Typically, football players start at level 2 for both parameters. After the demonstration training, the next difficulty level for each player should be set based on the following reasoning: (i) if a player achieves a score above 17 in any parameter (approximately an 80% success rate), the difficulty level should be increased by one; and (ii) if a player achieves a score below 6 or 7 (approximately a 30% success rate), the difficulty level should be decreased by one. It is important not to modify the difficulty levels after every session but to maintain them stable throughout the pilot phase. Ideally, each player should sustain a success rate between 30% and 80% for each parameter to maximize the effectiveness of the training [10,13,14]. The artifact handling (eye blinks, muscle noise) performed by iBrain software α i-Brain-Tech (https://ibrain.software/, accessed on 2 January 2024). The thresholds were individualized at 1 SD above baseline SMR, adjusted progressively. Prior studies showing effective SMR modulation within 20–30 min sessions [31].

The assessed variables were power, representing the participant’s ability to make quick tactical decisions or demonstrate technical actions under time constraints. Accuracy indicates how precise the participant’s decisions/actions were in alignment with the optimal tactical and technical responses. Goals, percentage of successful decisions/actions that directly or indirectly aligned with achieving a goal (e.g., scoring, advancing play effectively). Power DL (Difficulty Level), the complexity of tactical scenarios requiring quick decision-making or technical execution. Higher values suggest greater difficulty in decision-making under pressure. Accuracy DL (Difficulty Level), the complexity or ambiguity in scenarios requiring precise decisions or actions. Higher values imply more challenging situations where multiple options may seem viable. Power Sum, aggregated or weighted contribution of tactical decision-making speed and technical execution across all tasks, potentially normalized to account for difficulty. Accuracy Sum, aggregated or weighted contribution of tactical and technical precision across all tasks, also normalized for difficulty.

#### 2.3.3. Technical and Physical Performance

Performance metrics were evaluated using standardized protocols, including the LSST and the YYIR1, both recognized for their reliability in assessing athletic performance. The LSST is designed to assess a player’s shooting accuracy and proficiency under conditions that mimic match play [23,24]. During the test, players are required to execute a series of shots on goal from designated positions, aiming to hit specific targets within the goal area. The test is structured to evaluate both precision and decision-making speed. Performance is quantified based on the number of successful target hits and the time taken to complete the sequence. This test has been validated for use with female players, demonstrating reliability in distinguishing skill levels among participants [39,40]. The final variables evaluated were: LSST, these represent results from decision-making tasks where penalties or time delays reflect tactical and technical inefficiencies. LSST reflects performance in simulated scenarios, focusing on reaction time, average speed, and execution velocity in technical-tactical actions.

The YYIR1 evaluates an athlete’s ability to repeatedly perform high-intensity aerobic activities with short recovery periods, mimicking the intermittent demands of football [23]. The test consists of consecutive 2 × 20-m shuttle runs at progressively faster speeds, separated by brief recovery intervals. Only YYIR1 distance was used as a variable, without direct VO_2_max estimation. The total distance completed before the athlete reaches exhaustion is used as the performance metric. Although widely utilized in football to assess fitness levels, research suggests that the YYIR1 may not provide an accurate estimation of VO_2_max in women football players, highlighting the need for gender-specific testing protocols [23,41].

#### 2.3.4. Tactical Performance

Tactical performance was analyzed using a FUT-SAT-based instrument, which quantified correct and incorrect actions to assess motor efficiency and decision-making. The field test used in this system is called the “GK + 3 vs. 3 + GK” SSG format carried out for 4 min on a pitch measuring 36 m in length and 27 m in width. Each player performs the test twice, with a 2-min interval between repetitions. The matches are recorded by a video camera placed in a fixed elevated position, eliminating the need for equipment movement. The field dimensions were defined according to the official measurements established by the International Football Association Board (IFAB) and based on the ratio of space usage by outfield players. The duration was determined through a pilot study, which showed that 4 min was sufficient for players to execute all actions related to the tactical principles assessed by the observation instrument [14]. When compared to matches lasting up to 8 min. For the test, participants were randomly divided into two teams of three players, numbered 1 to 3 on one team and 4 to 6 on the other, to facilitate player identification in the video. During the games, players were instructed to follow the official laws of football, except for the offside rule, and recordings were made using a camera positioned diagonally in relation to the goal line and the sideline. In consequence this, the FUT-SAT-based instrument was adapted to small-sided conditions [26,28]. The final evaluated variables were: MEI encompasses various dimensions of tactical performance, including MEI Penetration, which measures the ability to make effective decisions for breaking through defensive lines; MEI Offensive Coverage, which evaluates support for the offensive unit through decision-making and positioning; MEI Depth Mobility, assessing off-ball movement to optimize depth in attacks; MEI Space, measuring the creation and utilization of space; MEI Offensive Unit, which reflects overall efficiency in offensive decision-making; MEI Delay, which indicates effectiveness in disrupting opponent transitions; MEI Defensive Coverage, evaluating decisions that bolster defensive structure; MEI Balance, assessing maintenance of tactical equilibrium in both attack and defense; MEI Concentration, reflecting focus in decision-making; and MEI Defensive Unit, capturing overall defensive efficiency. Similarly, DM metrics include DM Pen, evaluating decision-making related to penetration strategies; DM OC (Offensive Coverage), reflecting support for the offensive unit; DM Mobility, assessing movement-related positioning; DM Space, measuring spatial efficiency; DM Offensive Unit, a composite score for offensive decision-making; DM Delay, gauging efficiency in delaying transitions; DM DC (Defensive Coverage), evaluating defensive decisions; DM Balance, reflecting positional equilibrium; DM Concentration, highlighting critical focus in decision-making; and DM Defensive Unit, summarizing defensive decision-making performance. Additional variables include the DMI, which combines tactical decision-making scores, and Performance, representing overall tactical and technical efficiency.

### 2.4. Statistical Analysis

The statistical analysis was performed using Python (version 3.9) with the Pandas and Seaborn libraries for data manipulation and visualization [42,43]. Descriptive statistics included means, standard deviations, and minimum and maximum values. The normality of the data was verified using the Shapiro–Wilk test. A post hoc power analysis was conducted to evaluate the sensitivity of the correlational analyses performed. The statistical power (1 − β) for the Pearson’s correlation was calculated based on the sample size and the observed correlation coefficients from the dataset. Additionally, an a priori estimation of the required sample size to achieve a statistical power of 0.80 (with α = 0.05, two-tailed) was performed using the same analytical framework adopted in G*Power, Version 3.1.5.1 (Institut für Experimentelle Psychologie, Düsseldorf, Germany). Pairwise correlations between variables were computed using Pearson’s correlation coefficient (*r*) associated with 95% confidence intervals (95% CI). The correlation magnitude was classified as: trivial if *r* ≤ 0.1, small if *r* = 0.1–0.3, moderate if *r* = 0.3–0.5, large if *r* = 0.5–0.7, and very large if *r* = 0.7–0.9 and almost perfect if *r* ≥ 0.9. The significance of correlations was determined at *p* < 0.05. Variables with significant correlations were visualized in a heatmap for clarity, emphasizing strong associations within the dataset. The graphical visualization of the correlations was performed using a correlation heatmap generated with seaborn (heatmap function) and matplotlib (pyplot) in Python. Pearson’s correlation coefficients were computed to assess linear associations between continuous variables. The heatmap was configured with cmap=“RdBu_r” to represent correlation strength on a blue-to-red scale, and annot=True was applied to display numerical *r* values within each cell. Visualization limits were set with vmin=0.5 and vmax=0.9 to enhance contrast, and the figure size was defined as plt.figure(figsize=(10, 8)) to ensure clarity. A colorbar was included to indicate the scale, and the figure was titled “Heatmap of Pairwise Correlations (Corrected)” for clear and standardized presentation.

## 3. Results

Table 2 presents the descriptive statistics for all variables, including mean, standard deviation (SD), minimum, and maximum values. This provides an overview of the distribution and variability within the dataset.

Table 3 presents the significant pairwise correlations between key performance variables, including neurofeedback measures, MEI, DM, and technical and physical performance metrics. The correlations (*r*) provide insights into the strength and direction of the associations, and the *p*-values reveal the statistical significance. Strong correlations highlight associations between neurofeedback power, tactical success, DM efficiency, and overall performance. Table 2 presents the pairwise correlations between key variables, including demographic data, physical metrics, decision-making, motor efficiency indices, and technical/tactical variables. Significant correlations (*p* < 0.05) are highlighted to emphasize key associations.

**Figure 1:**
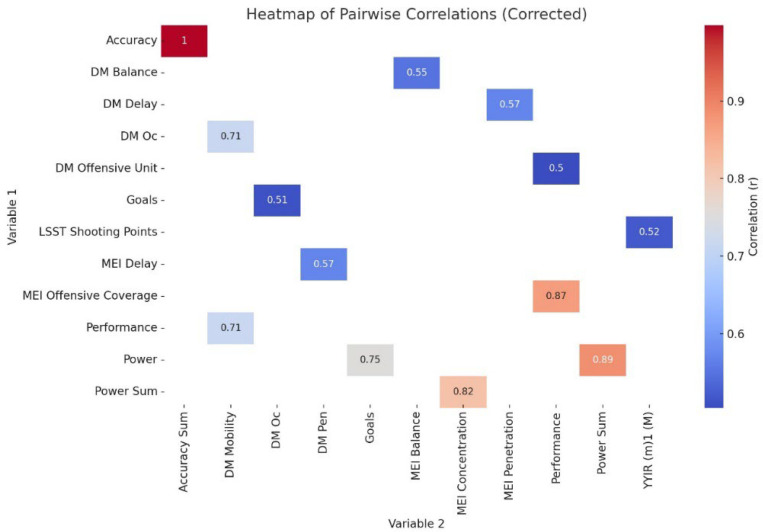
Heatmap of significant associations between Neurofeedback values, motor efficiency index, decision-making index, and technical performance.

The heatmap visualizes significant pairwise correlations between neurofeedback measures, MEI, DM, technical performance, and physical performance variables in youth female football players. The intensity of the colour represents the strength and direction of the correlations, with stronger positive correlations indicated by darker shades and weaker correlations represented by lighter shades (Figure 1).

## 4. Discussion

This study examined the associations between neurofeedback training outcomes and anthropometric, physical, technical, and tactical performance variables in young football players. The results demonstrated significant correlations between neurofeedback-derived he results demonstrated significant correlations between neurofeedback-derived indices and key performance measures, supporting the central hypothesis that enhanced neurophysiological regulation is linked to improved tactical decision-making, technical execution, and physical performance. These findings are consistent with previous evidence indicating that sensorimotor rhythm.

### 4.1. Neurofeedback, Power Efficiency, and Tactical Performance

The observed associations between neurofeedback power and tactical DM metrics suggest highlight the role of neural efficiency in supporting cognitive control and attentional stability during dynamic game scenarios. Players exhibiting higher power efficiency demonstrated improved tactical precision and reaction speed, aligning with previous studies showing that neurofeedback targeting SMR enhances cognitive adaptability and motor imagery in sport contexts [20,23,28]. Similar findings were reported in EEG-based and near-infrared neurofeedback research, where increased self-regulation of cortical rhythms correlated with improved tactical behavior and technical outcomes [11,12,40]. This aligns with evidence suggesting that SMR-based neurofeedback enhances attentional control, motor precision, and cognitive stability in athletes across sports domains [28,32,33]. The strong correlations between power sum and MEI concentration reinforce that sustained attentional focus facilitates tactical clarity and consistency across multiple contexts. Comparable results have been documented in studies linking executive functions and match performance in elite and youth football players [1,3,30,39,41].

Similarly to results reported in EEG-based and near-infrared neurofeedback studies [44,45], the players who demonstrated higher neurofeedback efficiency also showed improved indicators of tactical and technical execution. This neurophysiological-cognitive interaction is essential for supporting decision-making efficiency and tactical adaptability, particularly in high-pressure contexts typical of competitive football. Previous studies have reported that athletes who can effectively modulate cortical rhythms show enhanced sensorimotor integration and cognitive adaptability, allowing for more stable performance during complex decision-making scenarios [46,47]. Furthermore, the positive association between neurofeedback power and goals underscores the practical relevance of cognitive speed and precision for match effectiveness, aligning with evidence relating executive functioning to goal-oriented actions [29,40,45]. Current study associations reinforce the potential of neurofeedback as a complementary method for training cognitive readiness and self-regulatory capacity in youth football players [35,48].

The strong correlation between neurofeedback power sum and MEI concentration suggests that players with greater power efficiency maintain better focus, enabling more precise decisions in high-pressure scenarios. The positive association between neurofeedback power and goals emphasizes the importance of tactical decision speed in achieving successful outcomes, such as scoring. Lastly, the robust connection between neurofeedback power and power sum reflects the consistency required in applying tactical decisions across tasks, reinforcing the player’s overall execution efficiency. These findings collectively underline that power, as a measure of tactical speed and efficiency, directly supports decision-making focus and successful outcomes in soccer. The correlation between neurofeedback power and executive functions suggests that players exhibiting greater power efficiency are better able to maintain focus, which is critical for making precise decisions under pressure [32,45]. Furthermore, while the association between neurofeedback power and goals scored is not directly established in the literature, the significance of tactical decision speed in achieving successful outcomes, such as scoring, is emphasized in studies on executive functioning in football players [7,32]. This is further reinforced by the connection between executive functions and performance, indicating the consistency required in applying tactical decisions across various tasks, which enhances overall execution efficiency [44,45]. Collectively, these findings highlight that power, as a measure of tactical speed and efficiency, is integral to supporting decision-making focus and achieving successful outcomes in football [46,47]. Also, current research reinforces that neurofeedback represents a neurophysiological correlation of tactical speed and precision, supporting the integration of cognitive training methods to complement tactical development.

### 4.2. Aerobic Endurance and Technical Execution

The moderate positive correlation between LSST Shooting Points and YYIR1 highlights the link between aerobic endurance and technical consistency under fatigue. Aerobic endurance supports sustained physical and cognitive performance by delaying fatigue, critical for maintaining precision in high-pressure, game-like scenarios. This link highlights the interplay between physical fitness and technical skill execution, where improved endurance enables players to maintain composure and accuracy even under physically demanding conditions. The moderate positive correlation between LSST shooting points and YYIR1 distance indicates that players with superior aerobic endurance, as assessed by the YYIR1, are more capable of maintaining shooting accuracy under pressure during the LSST. These results are in line with prior studies demonstrating that greater aerobic capacity supports the maintenance of technical execution quality, such as shooting accuracy, during intense or prolonged match scenarios [13,32,38,43]. This association emphasizes that aerobic conditioning not only delays fatigue but also preserves cognitive processing efficiency, a key factor in decision accuracy during match play [5,44]. Previous research has similarly demonstrated that endurance capacity plays a critical role in sustaining technical performance throughout competitive matches, especially in female athletes [36,37,38]. Aerobic endurance is crucial for sustaining both physical and cognitive performance, as it helps delay fatigue, which is essential for maintaining precision in high-pressure, game-like scenarios [35,48]. The aerobic fitness positively influences technical consistency and passing or shooting accuracy during match play. Similarly, Almeida et al. [35] highlighted that aerobic endurance delays cognitive fatigue, which is essential for maintaining decision-making and precision throughout the game. This association underscores the interplay between physical fitness and technical skill execution, where enhanced endurance allows players to remain composed and accurate even in demanding conditions [48]. Furthermore, the ability to execute technical skills, such as shooting, is significantly influenced by a player’s physical fitness level. Studies have shown that improved aerobic capacity is linked to better performance in technical tasks, as players with higher fitness levels can execute skills more effectively during matches [8]. This connection highlights the importance of integrating aerobic training into football practice regimens to optimize both physical conditioning and technical skill performance [49]. Ultimately, the findings suggest that enhancing aerobic endurance can lead to improved technical execution, particularly in high-stakes situations where accuracy is paramount. Furthermore, the current findings thus emphasize the interplay between aerobic conditioning and technical skill, suggesting that endurance development contributes to sustained technical performance in match-relevant scenarios.

### 4.3. Decision-Making, Tactical Execution, and Cognitive-Motor Integration

The significant associations between DM Balance, MEI Balance, and performance indicators illustrate that equilibrium in decision-making aligns with tactical adaptability. This means that players who display balanced cognitive strategies are better at maintaining positional harmony between offensive and defensive actions, facilitating fluid transitions and team coordination. Players who maintain decision-making balance are better positioned to coordinate offensive and defensive actions, supporting fluid tactical transitions and collective team behavior. The associations emphasize the critical link between decision-making, tactical execution, and overall performance in football. The positive correlation between DM Balance and MEI Balance suggests that players who achieve equilibrium in their decisions are better at maintaining tactical balance, which is essential for effective transitions between offensive and defensive phases. These findings are consistent with evidence showing that tactical understanding, cognitive control, and spatial awareness are essential for optimizing performance [19,46,47]. The strong association between MEI Offensive Coverage and Performance highlights how precise decision-making and positioning in offensive plays directly enhance a player’s overall contribution to team success. These results are aligned with studies emphasizing the integration of perceptual-cognitive skills and tactical decision-making in football, especially in youth development contexts [17,18,48]. Similarly, the correlation between DM Mobility and Performance underscores the importance of movement-related positioning, where dynamic adaptability supports tactical and technical execution. Collectively, these findings justify the integral role of cognitive and spatial skills in optimizing football performance. The intricate association between DM, tactical execution, and performance in football is underscored by various studies. The positive correlation between DM Balance and MEI Balance indicates that players who maintain equilibrium in their decision-making are more adept at tactical balance, which is crucial for seamless transitions between offense and defense [28]. Furthermore, the strong link between MEI Offensive Coverage and overall performance emphasizes that precise decision-making and positioning during offensive plays significantly enhance a player’s contribution to team success [50,51]. Similarly, the association between DM Mobility and performance highlights the necessity of dynamic adaptability, where effective movement supports both tactical and technical execution [50,52].

Collectively, these findings affirm the essential role of cognitive and spatial skills in optimizing football performance, as they facilitate better anticipation, positioning, and execution of plays [28,53].

### 4.4. Practical Applications, Study Limitations, and Future Research

While the 6-week neurofeedback intervention yielded meaningful associations across multiple performance domains, it is important to acknowledge that these effects may reflect short-term adaptations. This study is among the first to examine the associations between neurofeedback metrics and multiple performance domains—anthropometric, physical, technical, and tactical—in youth female football players. A key strength is the multidimensional correlational design, allowing for a more integrated interpretation of cognitive, physical, and tactical interactions. However, the small sample size limits generalizability and statistical power. The exclusive focus on female youth athletes, while scientifically valuable, may not fully reflect broader populations, including male or mixed cohorts. Furthermore, the study’s short duration precludes conclusions regarding long-term adaptations to neurofeedback. Also, the research study design does not allow for the assessment of the long-term effects of neurofeedback training on performance; therefore, future research should adopt longitudinal designs to evaluate sustained impacts. The reliance on specific neurofeedback parameters suggests exploring alternative neurofeedback protocols to optimize outcomes. Longer-term or longitudinal interventions could help determine the persistence and consolidation of neurofeedback-induced improvements. Future research should also expand on the sample size and include male and mixed cohorts to improve external validity. Additionally, exploring different neurofeedback protocols beyond SMR (e.g., theta/beta ratio, alpha coherence) may help identify more specific cognitive mechanisms linked to football performance. Integrating neurofeedback with perceptual-cognitive or tactical training could further elucidate its potential as a complementary tool in player development. In addition, there is a lack of a control group and the short-term nature of the intervention (this study is part of a pre- and post-intervention study, but this correlational analysis only reports on the first phase, carried out pre-intervention, and is intended to be a correlational study). Lastly, integrating neurofeedback with diverse training methodologies could provide a more comprehensive understanding of its role in enhancing football performance. These directions could help establish evidence-based practices for neurofeedback in sports training. Future research should include larger and more diverse samples, use randomized controlled designs, and explore longitudinal interventions to assess the persistence of neurofeedback-induced effects. Integrating other neurofeedback protocols (e.g., theta/beta ratio, alpha coherence) may help uncover specific neural mechanisms underlying tactical behavior. Additionally, combining neurofeedback with tactical or perceptual-cognitive training may offer synergistic effects on performance. Emerging approaches using artificial intelligence and machine learning [1,54] may enhance the precision of EEG data interpretation and provide individualized training feedback.

## 5. Conclusions

This study provides evidence that SMR-based neurofeedback training is associated with improvements in cognitive and performance-related domains in female youth football players. The main findings indicate that neurofeedback Power relates to attentional focus, decision-making efficiency, and tactical skills in dynamic match contexts. Additionally, aerobic endurance was linked to technical stability under fatigue, while decision-making balance and mobility correlated with overall performance consistency. Anthropometric and maturational variables, although not directly predictive, may modulate neurofeedback responsiveness and training outcomes, suggesting that individual developmental status should be considered in future applications. By targeting neural efficiency and cognitive control, neurofeedback may enhance players’ readiness and adaptability—core determinants of high-level football performance.

## Figures and Tables

**Table 1:** Descriptive Statistics of anthropometric variables and experience level.

Variable	Mean ± SD
Age (years)	13.75 ± 1.77
Body Mass (kg)	52.86 ± 10.39
Standing Height (cm)	159.95 ± 6.65
Seated Height (cm)	130.80 ± 4.01
Experience level (years)	3.65 ± 1.98

**Table 2:** Descriptive statistics of neurofeedback, anthropometrics, technical, physical, and tactical performance in sampled young women’s football players.

Variable	Mean ± SD	Min	Max	Variable	Mean ± SD	Min	Max
Power	0.74 ± 0.15	0.50	0.95	DM Offensive Unit	0.68 ± 0.12	0.48	0.85
Accuracy	0.52 ± 0.10	0.35	0.72	DM Delay	0.60 ± 0.13	0.38	0.80
Goals	0.37 ± 0.08	0.25	0.55	DM DC	0.62 ± 0.14	0.40	0.85
Power Sum	0.40 ± 0.12	0.20	0.60	DM Balance	0.61 ± 0.13	0.42	0.82
Accuracy Sum	0.18 ± 0.05	0.12	0.30	DM Concentration	0.67 ± 0.14	0.45	0.88
MEI Penetration	0.68 ± 0.12	0.50	0.90	DM Defensive Unit	0.64 ± 0.13	0.45	0.85
MEI Offensive Coverage	0.62 ± 0.15	0.40	0.85	DMI	0.73 ± 0.11	0.50	0.90
MEI Depth Mobility	0.66 ± 0.14	0.45	0.85	Performance	0.71 ± 0.10	0.50	0.85
MEI Space	0.70 ± 0.13	0.48	0.88	Age	22.5 ± 2.1	18.0	26.0
Offensive Unit	0.65 ± 0.10	0.50	0.80	Body Mass (kg)	70.2 ± 7.5	55.0	85.0
MEI Delay	0.61 ± 0.14	0.40	0.82	Standing Height (cm)	175.4 ± 6.2	160.0	188.0
MEI Deffensive Coverage	0.59 ± 0.13	0.38	0.80	Sitting Height (cm)	90.5 ± 3.5	84.0	97.0
MEI Balance	0.63 ± 0.12	0.42	0.82	Leg Length (cm)	85.0 ± 5.0	75.0	92.0
MEI Concentration	0.67 ± 0.14	0.45	0.88	Maturity Offset	2.3 ± 0.5	1.5	3.5
MEI Deffensive Unit	0.60 ± 0.12	0.40	0.80	LSST_Total Time (s/+ with Penalties) T1	72.3 ± 5.2	62.0	80.5
MEI	0.64 ± 0.13	0.45	0.85	LSST_Total Time (s) Mean	10.2 ± 0.6	9.5	11.5
DM Pen	0.70 ± 0.14	0.45	0.90	LSST_Shot Speed (km/h) Mean	35.5 ± 6.3	25.0	45.0
DM OC	0.62 ± 0.15	0.40	0.85	LSST_Shot Points (mean of 10 shots) Mean	2.1 ± 0.4	1.5	2.8
DM Mobility	0.65 ± 0.14	0.42	0.85	YYIR1 (m)	560.0 ± 180.0	200.0	920.0
DM Space	0.63 ± 0.13	0.40	0.82				

Abbreviations: DM—Decision Making; OC—Offensive Coverage; Pen—Penetration; DC—Defensive Coverage; MEI—Match Evaluation Index; DMI—Decision Making Index; LSST—Loughborough Soccer Shooting Test; YYIR1—Yo-Yo Intermittent Recovery Test Level 1.

**Table 3:** Pairwise Correlations between neurofeedback, physical, decision-making, motor efficiency indices, and technical/tactical variables.

Variable 1	Variable 2	*r*	*p*
Power	Goals	0.75 **	<0.001
Power	Power Sum	0.89 **	<0.001
Accuracy	Accuracy Sum	0.99 **	<0.001
Goals	DM OC	0.51 *	0.020
DM OC	DM Mobility	0.72 **	<0.001
MEI Offensive Coverage	Performance	0.87 **	<0.001
MEI Delay	DM Pen	0.57 *	0.010
DM Offensive Unit	Performance	0.50 *	0.030
LSST_Finishing points	YYIR1 (m)	0.52 *	0.015
Power Sum	MEI Concentration	0.82 **	<0.001
DM Balance	MEI Balance	0.55 *	0.012
DM Delay	MEI Penetration	0.57 *	0.010
Performance	DM Mobility	0.72 **	<0.001

Abbreviations: DM—Decision Making; OC—Offensive Coverage; Pen—Penetration; MEI—Match Evaluation Index; LSST—Loughborough Soccer Shooting Test; YYIR1—Yo-Yo Intermittent Recovery Test Level 1. * *p* < 0.05; ** *p* < 0.001.

## Data Availability

The raw data supporting the conclusions of this article will be made available by the authors on request.
